# Riboflavin Targets the Cellular Metabolic and Ribosomal Pathways of Candida albicans *In Vitro* and Exhibits Efficacy against Oropharyngeal Candidiasis

**DOI:** 10.1128/spectrum.03801-22

**Published:** 2023-01-10

**Authors:** Junwen Lei, Jian Huang, Caiyan Xin, Fangyan Liu, Jinping Zhang, Yuxin Xie, Yingyu Mao, Wenbi Chen, Zhangyong Song

**Affiliations:** a The Platform of Molecular Biotechnology, Public Center of Experimental Technology, School of Basic Medical Sciences, Southwest Medical University, Luzhou, People’s Republic of China; Universidade de Sao Paulo

**Keywords:** antifungal agent, *Candida* species, fungal metabolism, oropharyngeal candidiasis, riboflavin

## Abstract

Oropharyngeal candidiasis (OPC), which has a high incidence in immunocompromised and denture stomatitis patients, is commonly caused by Candida albicans infection and in some cases develops into disseminated candidiasis throughout the throat and esophagus, resulting in high mortality. New drugs are needed to combat OPC because of the limited treatment options currently available and increasing resistance to existing drugs. Here, we confirmed that riboflavin (RF), a cofactor of flavin adenine mononucleotide and flavin adenine dinucleotide, has broad-spectrum anti-*Candida* activity. The formation of C. albicans hyphae and biofilm was inhibited by RF. Mechanistically, RF disrupted membrane and cell wall integrity, as well as promoting reactive oxygen species and pyruvate accumulation. Furthermore, RF targeted multiple essential pathways via functional disruption of thiamine and RF metabolic pathways, central carbon metabolism, and ribosome metabolism. Similar to the results *in vitro*, the inhibitory effect of RF on C. albicans hyphae was confirmed in a mouse model of OPC. Moreover, after 5 consecutive days of intraperitoneal injection, RF exhibited therapeutic efficacy, as demonstrated by phenotype investigation, the fungal burden, and histopathological analysis. These findings revealed that RF exerts a multifaceted anti-*Candida* effect and has potential benefits in the treatment of OPC.

**IMPORTANCE** *Candida* species are common pathogens in fungal infections, causing mucosal infection and invasive infection in immunodeficient patients. Given the limited classes of drugs and resistance to these drugs, new antifungal agents need to be developed. Drug repurposing is a potential method for antifungal drug development. This study demonstrated that riboflavin (RF) exhibited broad-spectrum anti-*Candida* activity. RF affected multiple targets involving the membrane and cell wall integrity, the accumulation of reactive oxygen species and pyruvate, and the altered metabolic pathways in C. albicans. Moreover, RF exhibited efficacy in the treatment of C. albicans in an oropharyngeal candidiasis mouse model. Taken together, the antifungal activity and the promising clinical application of RF were highlighted.

## INTRODUCTION

Candida albicans is a common opportunistic fungal pathogen that causes superficial cutaneous and mucosal infections, as well as invasive infections, known as candidiasis and candidemia, respectively (1, 2). C. albicans symbiotically inhabits the oral cavity of healthy individuals. However, it can become pathogenic, causing oropharyngeal candidiasis (OPC) in immunocompromised individuals (especially those with AIDS, diabetes, cancer, or organ transplantation) (3). The exacerbation and recurrence of OPC provide favorable conditions for the occurrence of candidemia and increase the difficulty of treatment of the primary disease (4). In addition, the emergence of coronavirus has further increased the morbidity associated with *Candida* infection, including in OPC patients (5, 6).

At present, only four classes of drugs (azoles, polyenes, echinocandins, and flucytosine) are available for the treatment of pathogenic fungal infections. These agents target the fungal cell membrane, cell wall, or DNA/RNA (7). However, the widespread use of antifungal agents, as well as the limitation of drug classes and targets, has caused various levels of fungal resistance. The emergence of drug-resistant species, including Candida auris, has also further threatened the treatment of pathogenic fungi (8). Moreover, both fungi and human hosts are eukaryotes (9), which further complicates the development of uniquely targeted antifungal agents. The time and costs involved in the development of new classes of drugs have meant that no new antifungal agents have been approved in the 20th century (10, 11). Therefore, drug repurposing is gaining increased attention as an alternative method for antifungal drug development.

Riboflavin (RF), also known as vitamin B_2_, is an intracellular precursor of flavin adenine dinucleotide (FAD) and flavin mononucleotide (FMN) and is involved in fatty acid oxidation, methylation, mitochondrial electron transport, nucleotide synthesis, and redox reaction processes (12). Most mammals, including humans, are unable to synthesize RF. RF derived from the diet is absorbed and metabolized in the human gut (13). RF deficiency results in cleft lip, glossitis, and oral mucosal edema; it can also affect gastrointestinal and brain function, as well as the metabolism of other vitamins (such as folate, niacin, pyridoxine, and vitamin B_12_) (13). Besides its classic role, RF also has application prospects in the field of anti-infection (14). Since RF is a photosensitizer, RF-mediated photodynamic therapy has an inhibitory effect on microbial keratitis (14), as well as infections caused by Escherichia coli, Listeria monocytogenes, Plasmodium falciparum, Staphylococcus aureus, and dengue virus (15–19). Furthermore, the level of RF was reported to be reduced in patients infected with human papillomavirus 16/18 or Helicobacter pylori (20, 21). Moreover, RF has a synergistic effect with fluconazole (FCZ) in treating vaginal candidiasis (22). However, the direct mechanism of this anti-*Candida* effect and the application of RF in OPC have not been reported. Considering these previous promising studies, the *in vitro* and *in vivo* anti-*Candida* effects of RF were examined in this study. Our results suggested that RF has a broad-spectrum anti-*Candida* effect *in vitro* by generating reactive oxygen species (ROS) and targeting cellular metabolic and ribosomal pathways. Moreover, *in vivo*, RF reduced the fungal burden in an OPC murine model, and the adhesion to and invasion of oral epithelial cells by C. albicans were decreased after RF treatment.

## RESULTS

### Broad-spectrum activity of RF inhibits *Candida* growth *in vitro*.

**FIG 1:**
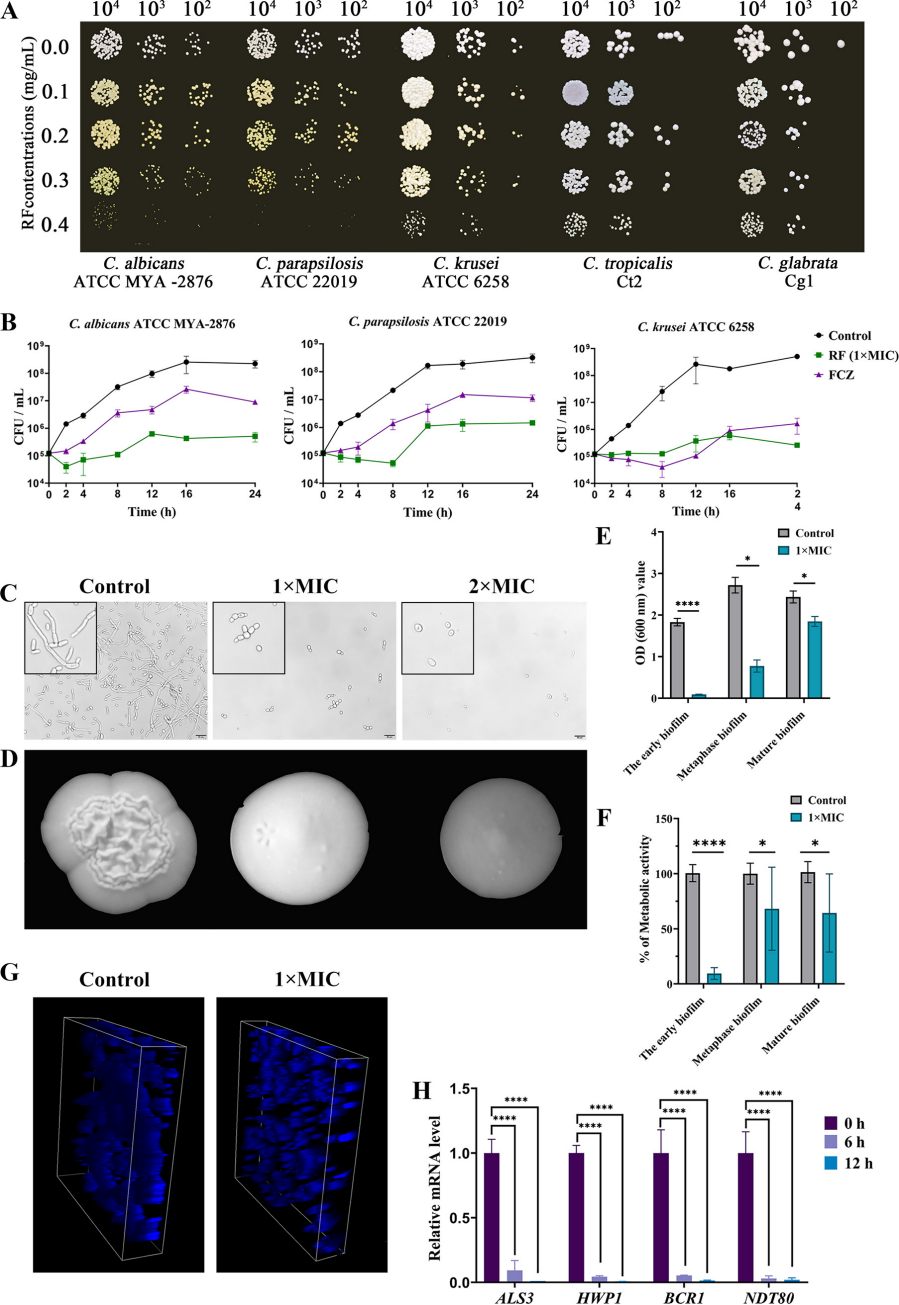
The inhibitory effect on cell growth and hyphal and biofilm formation of *Candida* species. (A) The growth of *Candida* species on YPD agar plates with different concentrations of RF. (B) Growth curves of standard strains C. albicans ATCC MYA-2876, C. krusei ATCC 6258, and C. parapsilosis ATCC 22019 treated with 0.4 mg/mL of RF. (C) Hyphal formation was evaluated in RPMI 1640 plus 10% (vol/vol) FBS liquid medium, and uniformly enlarged images are presented in the black boxes on the left side. Bar, 20 μm. (D) Hyphal formation was evaluated on YPD plus 10% (vol/vol) FBS agar plates. (E) The biomass of C. albicans biofilm was observed by a crystal violet assay. (F) Metabolic activity of C. albicans biofilm was determined by a 2,3-bis-(2-methoxy-4-nitro-5-sulfophenyl)-2H-tetrazolium-5-carboxanilide assay. The results are presented as relative percentages. (G) The three-dimensional structure of C. albicans biofilm was stained with calcofluor white and observed by confocal laser scanning microscopy. (H) The expression of biofilm-related genes was determined. C. albicans treated with 1× MIC of RF was incubated for 0, 6, and 12 h before RNA extraction. 1× MIC, 0.4 mg/mL of RF; 2× MIC, 0.8 mg/mL of RF. Data were analyzed by a *t* test or one-way analysis of variance (one-way ANOVA) (ns [not significant], *P* > 0.05; *, *P* < 0.05; **, *P* < 0.01; ***, *P* < 0.001; ****, *P* < 0.0001).

The MICs of RF for four standard strains and five clinical isolates were determined. RF exhibited broad-spectrum activity against *Candida* species, with an MIC of 0.4 mg/mL. With the dose of RF increased, the anti-*Candida* effect became more significant in a spot assay (Fig. 1A). A growth curve assay further characterized the fungicidal effects of RF. FCZ was used as a positive-control drug. Compared with the control group, 1× MIC of RF inhibited more than 90% of C. albicans, Candida krusei, and Candida parapsilosis grwoth between 2 and 24 h (Fig. 1B), and the antifungal activity of 1× MIC of RF was more effective than that of the FCZ group (64 μg/mL).

### RF inhibits *Candida* hypha and biofilm formation *in vitro*.

The formation of *Candida* hyphae plays an important role in the invasion and damage of epithelial and endothelial cells, as well as in causing bloodstream infections (23, 24). The effect of RF on C. albicans hyphal formation under a hypha-induced medium was elucidated. Hypha cells and both tubular and multicellular forms of C. albicans were inhibited by RF in liquid medium (Fig. 1C). The antihypha capacity of RF was further confirmed by the transition from crenulated to smooth colonies on solid medium (Fig. 1D).

Filamentous development is critical for biofilm formation, and biofilm formation on medical devices is the main cause of nosocomial fungal infections (25, 26). The effect of RF on biofilm formation was further evaluated. A crystal violet (CV) assay and a 2,3-bis-(2-methoxy-4-nitro-5-sulfophenyl)-2H-tetrazolium-5-carboxanilide (XTT; Macklin, Shanghai, China) assay were used to analyze the effect of RF on the formation, development, and maturity of biofilms. As shown in Fig. 1E and F, 1× MIC of RF seriously affects the early biofilm formation and has a minor effect on destroying the mature biofilm. To better define the effect of RF on biofilm formation, confocal laser scanning microscopy (CLSM; Leica, Beijing, China) investigation was performed and revealed that C. albicans cells were compact and connected into sheets in the absence of RF treatment. Conversely, cells treated with RF showed low density and could not connect into sheets (Fig. 1G). Subsequently, the transcription levels of genes related to hyphal and biofilm formation were evaluated by reverse transcription-quantitative PCR (RT-qPCR). Hyphal-formation-specific genes (including *ALS3*, *HGT2*, and *HWP1*) and a biofilm development-specific gene (*BCR1*) were analyzed. As expected, all genes were downregulated after RF treatment (Fig. 1H). Moreover, to further confirm the anti-hyphae and anti-biofilm activity against other *Candida* species, a similar effect was observed on C. parapsilosis ATCC 22019 hyphal and biofilm formation after RF treatment (see Fig. S1 in the supplemental material).

### RF treatment damages *Candida* cell wall integrity.

**FIG 2:**
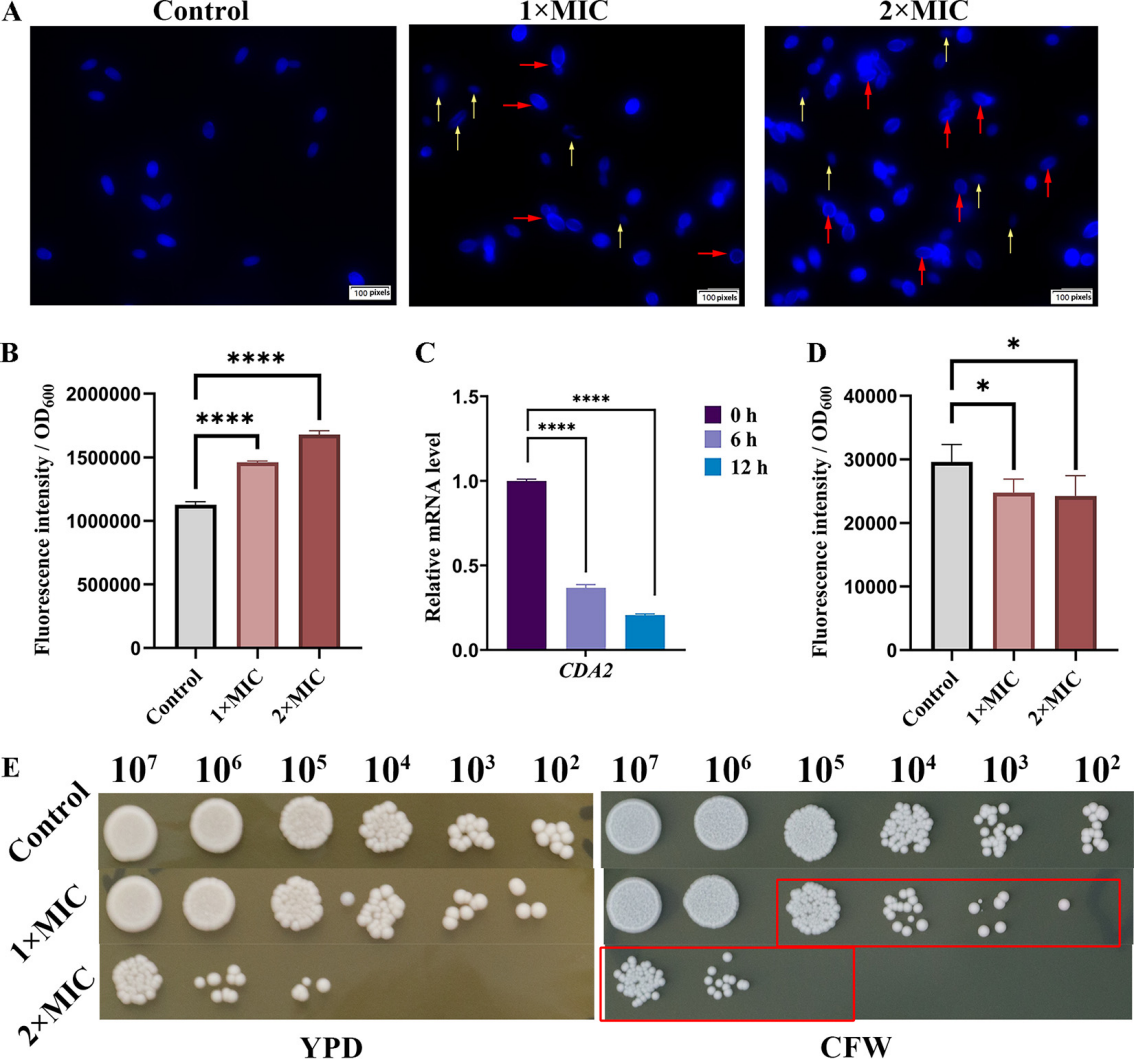
The effect of RF on the C. albicans cell wall. (A) C. albicans with or without RF treatment was stained with calcofluor white (CFW) and observed under a fluorescence microscope. CFW binds fungal chitin and produces blue fluorescence. Yeast cells with a disrupted elliptical structure are indicated by yellow arrows. Chitin deposition is indicated by red arrows. Images were captured at ×60 magnification using a fluorescence microscope. (B) Chitin levels were quantified through the fluorescence intensity of cells stained with CFW. (C) The expression of the gene encoding chitin deacetylase (*CDA2*) was determined. (D) Total glucan levels were quantified through the fluorescence intensity of cells stained with aniline blue. (E) C. albicans with or without RF treatment was spotted onto YPD agar containing 50 μg/mL of CFW and incubated for 24 h. Data were analyzed by one-way ANOVA (ns, *P* > 0.05; *, *P* < 0.05; **, *P* < 0.01; ***, *P* < 0.001; ****, *P* < 0.0001).

The fungal cell wall is involved in cell morphological maintenance and polarized growth (27). *Candida* can survive by altering its cell wall composition and structure in changed environments (28). As shown in Fig. 2A, calcofluor white (CFW; Sigma, Shanghai, China) was homogeneously distributed in yeast cells in the absence of RF treatment. However, more CFW was aggregated and distributed in the outer layer of C. albicans cells, especially at the budding end following RF treatment (red arrows). Even the elliptical structure of some yeast cells was disrupted (yellow arrows). Furthermore, investigation of CFW fluorescence indicated that the chitin content was increased by RF (Fig. 2B), and the expression of *CDA2* (encoding chitin deacetylase, which catalyzes chitin to chitosan [29, 30]) was obviously downregulated, implying that the increase in chitin occurred as a result of the blocked consumption pathway (Fig. 2C). In addition, the aniline blue staining assay showed that total glucan levels were decreased following RF treatment (Fig. 2D). Investigation of the susceptibility of C. albicans cells to RF under CFW stress conditions also showed that pretreatment with RF inhibited cell growth (Fig. 2E, red rectangle). These data suggest that RF remodels the C. albicans cell wall structure.

### RF treatment alters *Candida* cell membrane permeability.

**FIG 3:**
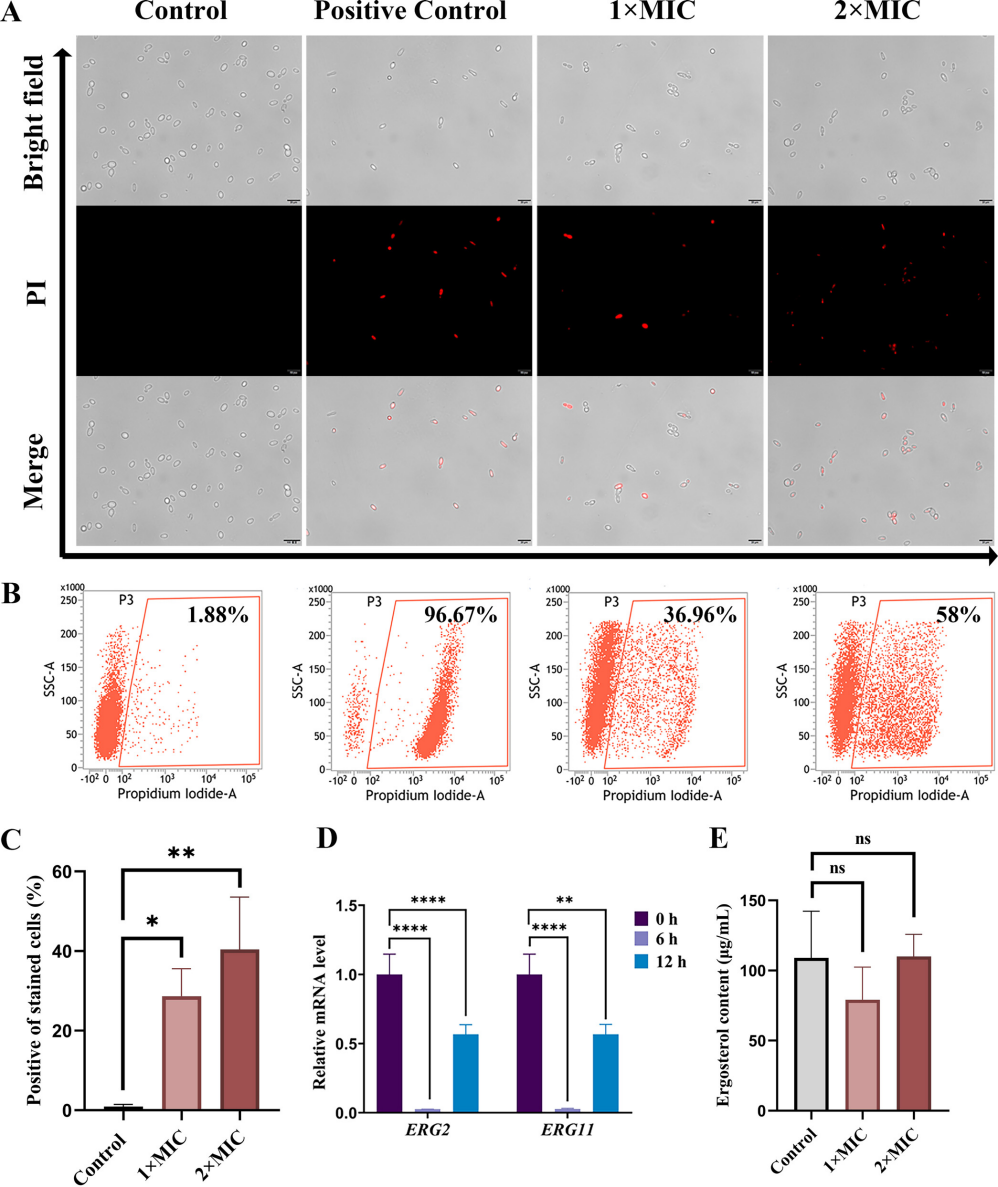
The effect of RF on the C. albicans cell membrane. (A and B) C. albicans cells with or without RF treatment were stained with propidium iodide (PI) and analyzed by fluorescence microscopy (A) and flow cytometry (B). Bar, 20 μm. DIC, differential inference contrast; SSC, side scatter. (C) Histogram analysis shows the percentage of PI-positive cells. (D) The expression of genes involved in ergosterol synthesis was determined. (E) Histogram analysis shows the ergosterol concentration of C. albicans cells. Data were analyzed by one-way ANOVA (ns, *P* > 0.05; *, *P* < 0.05; **, *P* < 0.01; ***, *P* < 0.001; ****, *P* < 0.0001).

The transport of RF has been reported to be associated with the cell membrane in Bacillus subtilis (25). To further explore the effect of RF on the structure of *Candida*, its cell membrane integrity was evaluated. Propidium iodide (PI) enters cells through a damaged cell membrane and binds to nucleic acids, producing red fluorescence. As shown in Fig. 3A, the number of red cells increased after RF treatment, while there were no red cells in the control group. Similarly, a flow cytometry assay further confirmed that 36.96% and 58.00% of C. albicans cells were stained fluorescently following RF treatment with 1× MIC and 2× MIC, respectively (Fig. 3B and C). It is worth noting that cells with damaged cell membranes cannot survive after being spread onto yeast extract-peptone-dextrose (YPD) agar (Fig. 1B). Additionally, there is a similar effect on the C. parapsilosis ATCC 22019 cell membrane (Fig. S2). Ergosterol, the main component of the *Candida* cell membrane, has become a focus of the development of antifungal drugs (31). In this study, the genes involved in the ergosterol synthesis pathway (including *ERG2* and *ERG11*) were analyzed by RT-qPCR. As shown in Fig. 3D, the expression of *ERG2* and *ERG11* was downregulated after RF treatment. However, compared with the control group, the content of ergosterol in C. albicans showed no statistically significant difference after treatment with 1× MIC and 2× MIC of RF (Fig. 3E). In short, RF enhanced cell membrane permeability without affecting the ergosterol content.

### RF treatment induces ROS accumulation.

**FIG 4:**
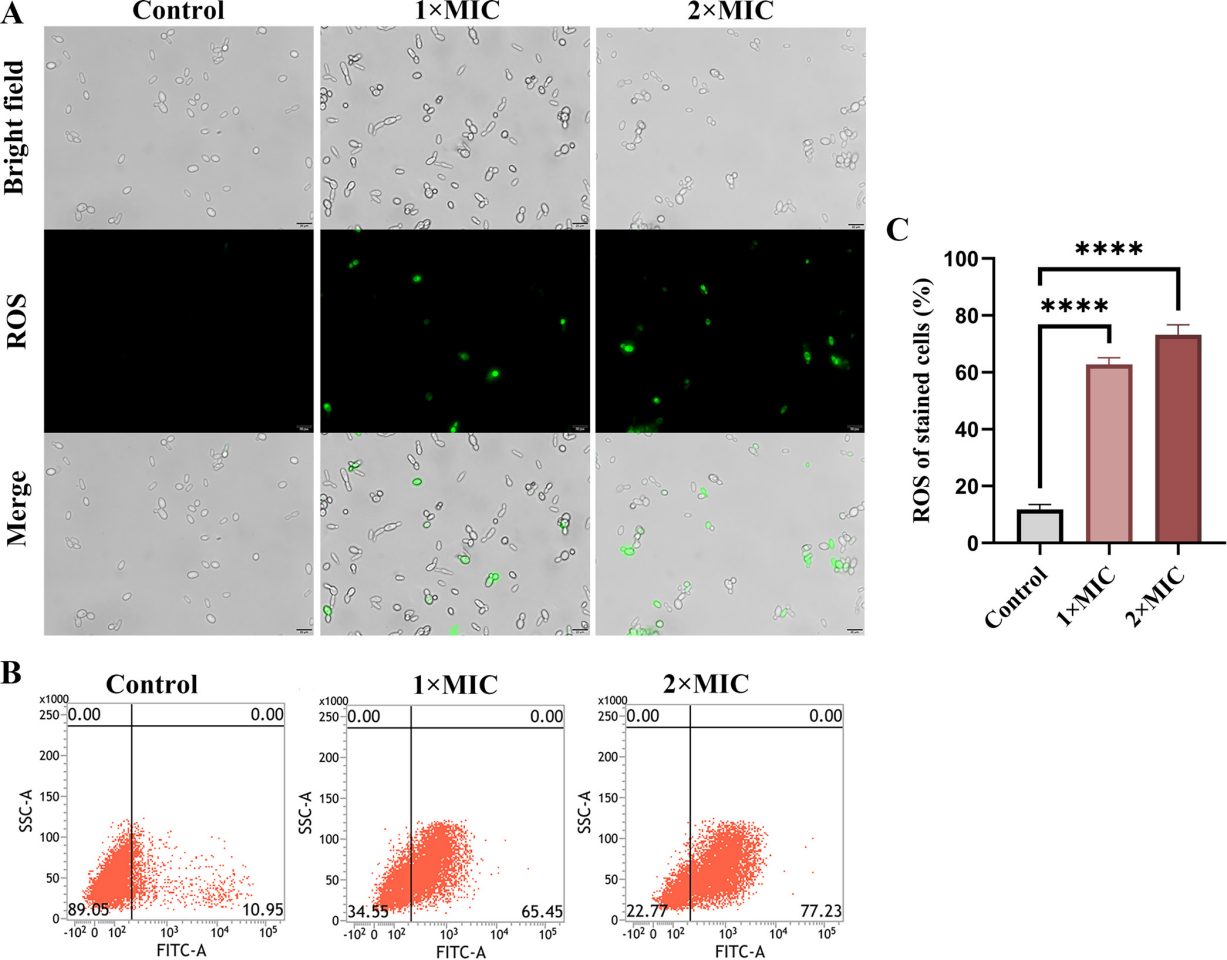
The effect of RF on reactive oxygen species (ROS) production. (A) The accumulation of ROS green fluorescence was observed. ROS oxidizes DCFH-DA to DCF, emitting green fluorescence. Bar, 20 μm. (B) The relative percentage of ROS-positive cells was shown by flow cytometry. FITC, fluorescein isothiocyanate. (C) Histogram analysis showed the percentage of ROS-positive cells. Data were analyzed by ANOVA (ns, *P* > 0.05; *, *P* < 0.05; **, *P* < 0.01; ***, *P* < 0.001; ****, *P* < 0.0001).

ROS are superoxides produced in mitochondria, and excessive levels of intracellular ROS affect the integrity of cell walls and cell membranes (32). Thus, ROS production was measured. As shown in Fig. 4A, significantly increased fluorescence was observed after treatment with RF. Flow cytometry further confirmed that 65.45% and 77.23% of cells were ROS positive after RF treatment with 1× MIC and 2× MIC, respectively, while only 10.95% of cells were ROS positive in the control group (Fig. 4B). These results suggested that RF markedly promotes the excessive production of ROS (Fig. 4C).

### RF induces coenzyme metabolic disorder.

**FIG 5:**
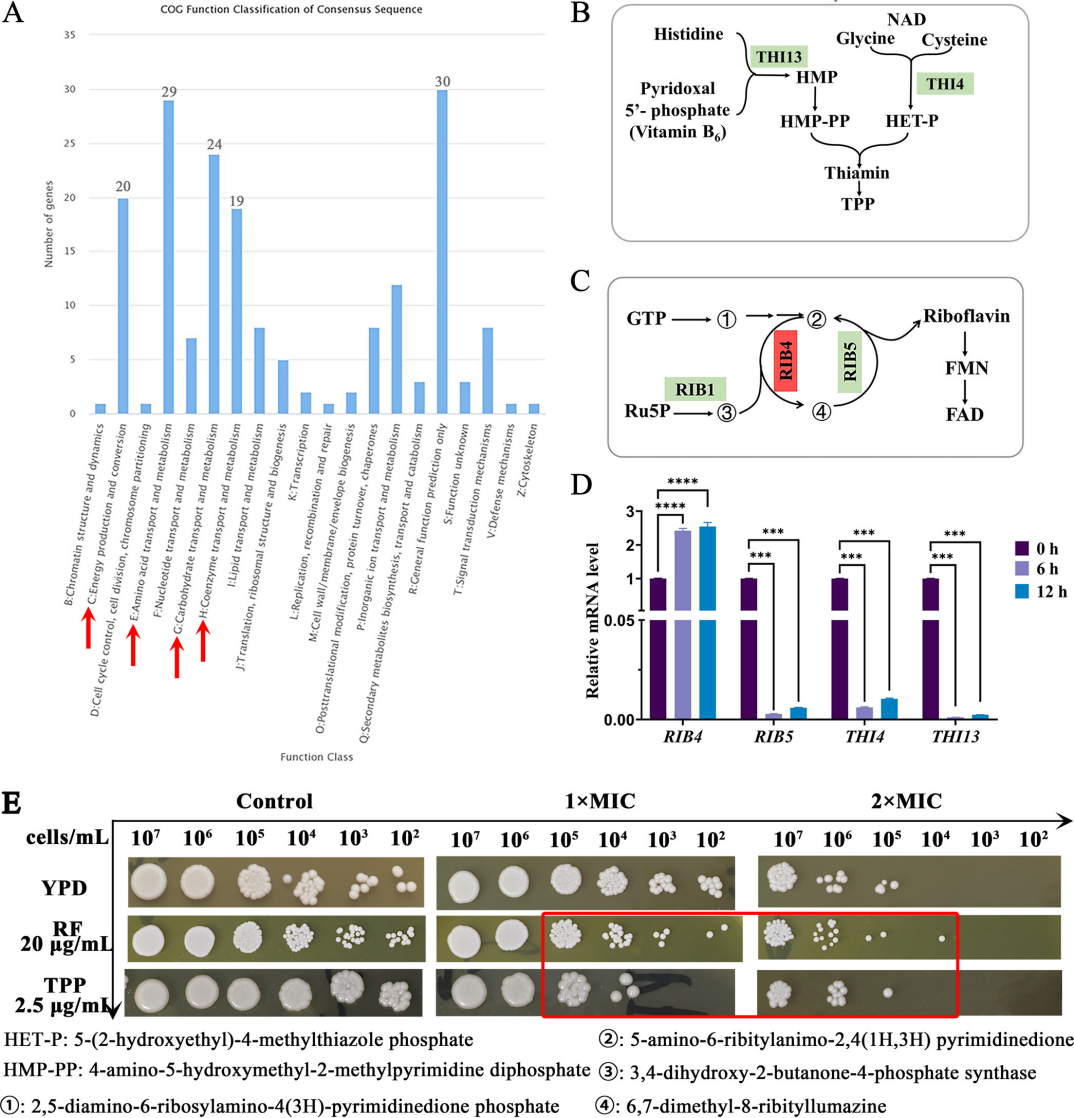
The effect of RF on C. albicans coenzyme metabolism. (A) Clusters of orthologous groups analysis of downregulated differentially expressed genes was performed, and the main enrichment pathways are indicated by red arrows. (B and C) Schematic model of the endogenous thiamine metabolism pathway (B) and the endogenous riboflavin metabolism pathway (C). Genes encoding proteins in red were upregulated, while genes encoding proteins in green were downregulated. (D) The expression of genes involved in the thiamine and RF metabolic pathways was analyzed. (E) C. albicans cells with or without RF treatment were spotted onto YPD agar plates containing exogenous thiamine or RF. TPP, thiamine pyrophosphate. Data were analyzed by ANOVA (ns, *P* > 0.05; *, *P* < 0.05; **, *P* < 0.01; ***, *P* < 0.001; ****, *P* < 0.0001).

The accumulation of ROS induces protein, lipid, and nucleic acid damage, resulting in C. albicans metabolic disorder and cell death (33). To analyze the mechanism of action of RF in greater depth, RNA-sequencing (RNA-Seq) was performed. The results showed that 563 genes were upregulated and 469 genes were downregulated. The downregulated differentially expressed genes were further analyzed and were found to be enriched in energy production and conversion, amino acid transport and metabolism, and carbohydrate transport and metabolism (Fig. 5A). Unexpectedly, genes involved in coenzyme transport and metabolism were most significantly altered, especially those in the coenzyme thiamine and RF metabolism pathways. Therefore, the expression of key genes in the thiamine (*THI4* and *THI13*, Fig. 5B) and RF (*RIB4*, and *RIB5*, Fig. 5C) metabolic pathways was subsequently analyzed. The results showed that exogenous RF significantly decreased the expression of *RIB5*, *THI4*, and *THI13*, while the expression of *RIB4* was increased by RF treatment (Fig. 5D). RF-treated C. albicans was spotted onto YPD medium containing thiamine pyrophosphate (TPP) and RF, which are the metabolites of the coenzyme metabolic pathway. As shown in Fig. 5E, the growth of RF-treated C. albicans was inhibited following the addition of TPP or RF stress (red rectangle). These data suggested that RF induces thiamine and RF metabolic disorder in C. albicans.

### RF affects central carbohydrate metabolism.

**FIG 6:**
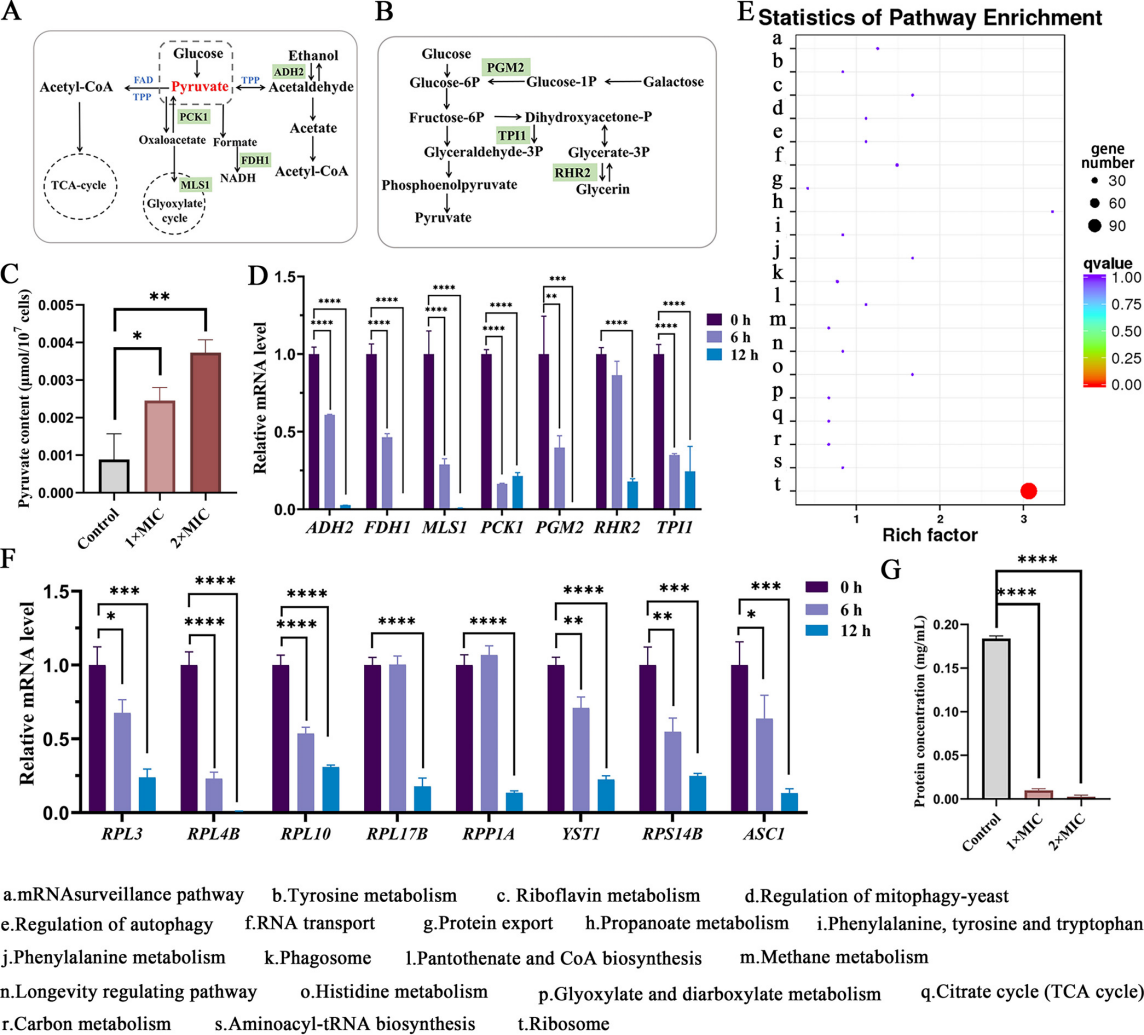
The effect of RF on C. albicans central carbon metabolism and ribosome metabolism. (A and B) A schematic model of central carbon metabolism (A) and glycometabolism (B). Genes encoding proteins in red were upregulated, while genes encoding proteins in green were downregulated. TCA, tricarboxylic acid. (C) The concentration of pyruvate was determined. (D) The expression of genes involved in central carbon metabolism was determined. (E) Kyoto Encyclopedia of Genes and Genomes analysis of upregulated differentially expressed genes. (F) The expression of genes involved in ribosome metabolism was determined. (G) Histogram analysis shows the concentration of total protein with or without RF treatment. 1× MIC, 0.4 mg/mL of RF; 2× MIC, 0.8 mg/mL of RF. Data were analyzed by ANOVA (ns, *P* > 0.05; *, *P* < 0.05; **, *P* < 0.01; ***, *P* < 0.001; ****, *P* < 0.0001).

Coenzymes FAD and TPP, as members of the pyruvate oxidative decarboxylase complex, are involved in the enzymatic conversion of pyruvate to acetyl coenzyme A (34, 35) (Fig. 6A and B). It has been confirmed that pyruvate accumulation is the first line of fungal cell defense against ROS caused by heat stress (36). Thus, the content of pyruvate was measured. As shown in Fig. 6C, the concentration of pyruvate was increased in C. albicans after treatment with RF. In addition, pyruvate is an important regulator of central carbohydrate metabolism in the cell, linking the glycolytic pathway to the tricarboxylic acid cycle (34). Suppression in central carbohydrate metabolism was confirmed by RNA-Seq (Fig. 5A) and the downregulation of related genes (including *ADH2*, *FDH1*, *MLS1*, *PCK1*, *PGM2*, *RHR2*, and *TPI1*) (Fig. 6D). These results implied that exogenous RF targets internal RF and the thiamine metabolic pathway, increasing the concentration of pyruvate, which in turn induces central carbohydrate metabolic disorders in C. albicans.

### RF affects ribosome metabolism.

The upregulated differentially expressed genes were also analyzed, and 134 out of 563 upregulated genes were enriched in ribosome metabolism (Fig. 6E). To confirm this finding, eight genes were selected for additional RT-qPCR analysis. Ribosomes consist of a large 60S subunit (encoded by *RPL3*, *RPL4B*, *RPL10*, *RPL17B*, and *RPP1A*) and a small 40S subunit (encoded by *YST1*, *RPS14B*, and *ASC1*). The expression of all of these genes was obviously downregulated following RF treatment for 6 or 12 h, with the exception of *RPP1A* (Fig. 6F). Ribosomes are responsible for reading mRNA to synthesize protein (37). Therefore, we measured the total protein content of C. albicans following RF treatment and found it to be significantly decreased (Fig. 6G), indicating that RF induced the dysfunction of ribosome metabolism.

### RF improved the symptoms of OPC *in vivo*.

**FIG 7:**
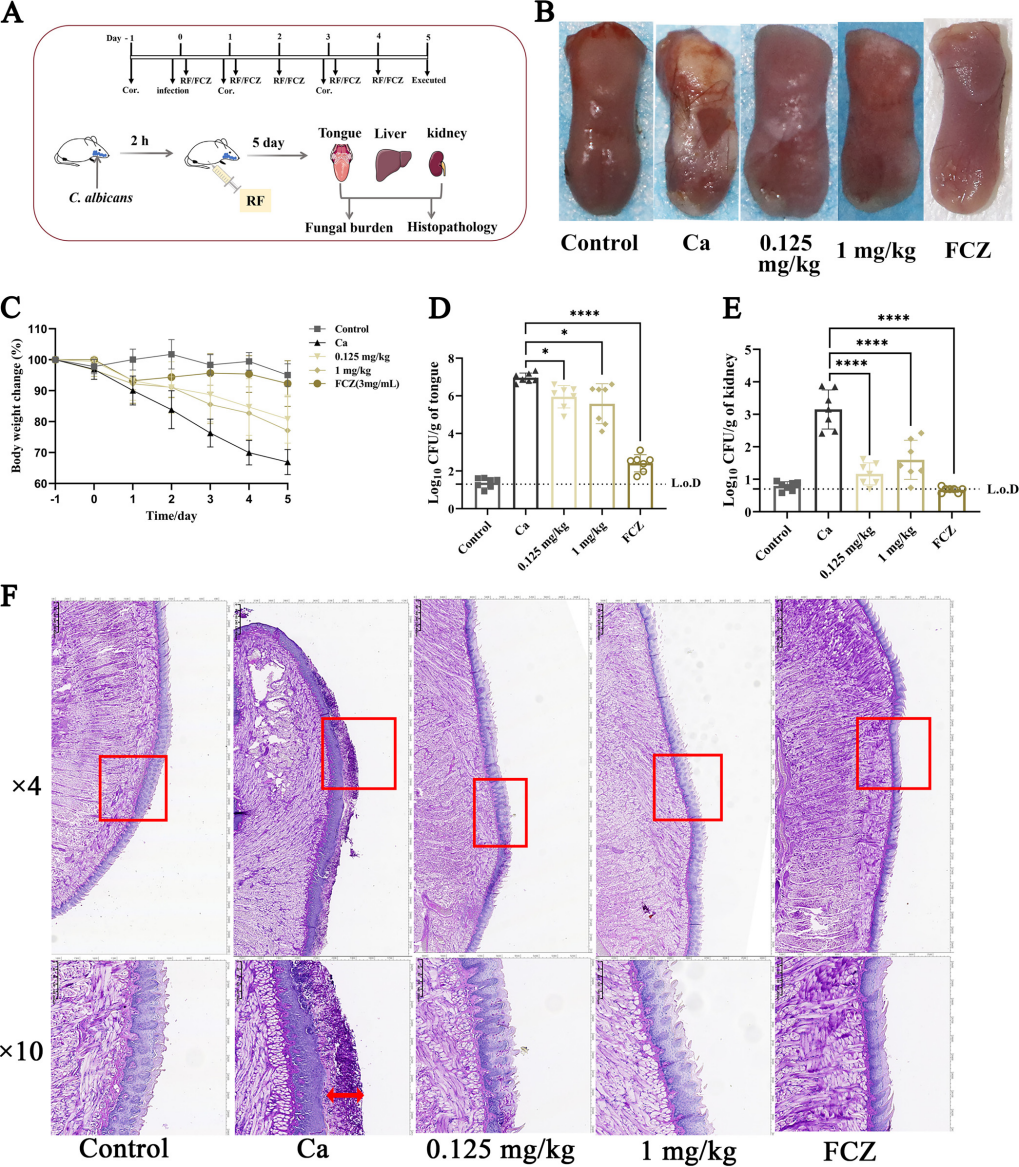
The effect of RF on an oropharyngeal candidiasis (OPC) mouse model. (A) A schematic model of the experiment. Mice were injected with immunosuppressant cortisone acetate on days −1, 1, and 3. Mice were infected with C. albicans and continuously treated with normal saline, RF, or fluconazole (FCZ) for 5 days. All mice were euthanized on day 5, and the tongue, kidney, and liver were collected. (B) Visual analysis of the phenotype of mouse tongues. Control group, immunodeficient and uninfected mice that were continuously treated with sterile saline; Ca group, immunodeficient and infected mice that were continuously treated with sterile saline; 0.125- and 1-mg/kg groups, immunodeficient and infected mice that were continuously treated with 0.125 and 1 mg/kg of RF, respectively; FCZ, immunodeficient and infected mice that were continuously treated with 13.6 mg/kg of FCZ. (C) Changes in the body weight of mice over 5 consecutive days. (D and E) Fungal burden on the tongue (D) and kidneys (E). L.o.D., limit of detection. (F) Histopathological analysis by periodic acid-Schiff staining. In the image showing an ×4 magnification, the area in the red rectangle is shown at ×10 magnification below. The damage to tongue epithelial cells caused by C. albicans is indicated by red arrows. Data were analyzed by ANOVA (ns, *P* > 0.05; *, *P* < 0.05; **, *P* < 0.01; ***, *P* < 0.001; ****, *P* < 0.0001).

The efficacy of RF *in vivo* was investigated in an OPC murine model. First, immunosuppressed mice were infected with C. albicans. After 2 h of incubation, RF or normal saline was injected intraperitoneally (Fig. 7A). As shown in Fig. 7B, the back of the tongue was ruddy and smooth in the control group, whereas a thick white plaque was evident on the dorsum of the tongue in the Ca group (OPC mice treated with normal saline). After administering 0.125 or 1 mg of RF per kg of body weight or 13.6 mg/kg of FCZ for 5 days, the white plaques were thinner and showed less coverage. In addition, the loss in body weight in the RF-treated group was less than that in the Ca group (Fig. 7C). Importantly, compared with the Ca group, there was a statistically significant reduction in the fungal burden on the tongue and kidneys in the RF-treated group (Fig. 7D and E); however, there was no statistical difference in the liver (data not shown). To assess the curative efficacy of RF *in vivo*, histopathological changes on the mouse tongue were evaluated by periodic acid-Schiff (PAS) staining. In the Ca group, the tongue epithelium covered a large number of hypha cells destroying the papillae (Fig. 7F). In contrast, fewer hyphal cells and thin and loose biofilm were found in the tongue epithelium of the RF-treated group. In addition, for the RF-treated group, damage of the tongue papillae was relieved (Fig. 7F). These results indicated that RF has an antifungal effect in an OPC murine model.

## DISCUSSION

The prevalence of novel pathogens has been on the rise over the past few decades, as evidenced by the emergence of novel coronavirus COVID-19/SARS-CoV-2, Ebola viruses, Middle East respiratory viruses, C. auris, and novel mutant bacteria (38–40). However, drug development has not kept pace with the rise of novel pathogens and the resistance to antimicrobial drugs, which provides an opportunity for drug repurposing (11). Drug repurposing is the search for new uses or characteristics from approved drugs, such as molecular targets, mode of action, or pharmacological effects (11). Such a strategy reduces the risk, time taken, and cost expended on new drug development. Recent studies have emphasized the important role of B vitamins in the human nervous system, immune response, and tumor development (41–43). In addition, B vitamins also have value in patients with kidney disease, sepsis, and COVID-19 (44–46). Our previous review summarized the anti-infection effect of RF and suggested that RF may have direct antimicrobial effects (14). Here, *in vitro* and *in vivo* phenotypic investigations confirmed that RF has anti-*Candida* ability. *In vitro*, RF is able to inhibit biofilm formation. Adhesion, which is the first step in biofilm formation, was also found to be reduced after RF treatment (see Fig. S3A and B in the supplemental material).

**FIG 8:**
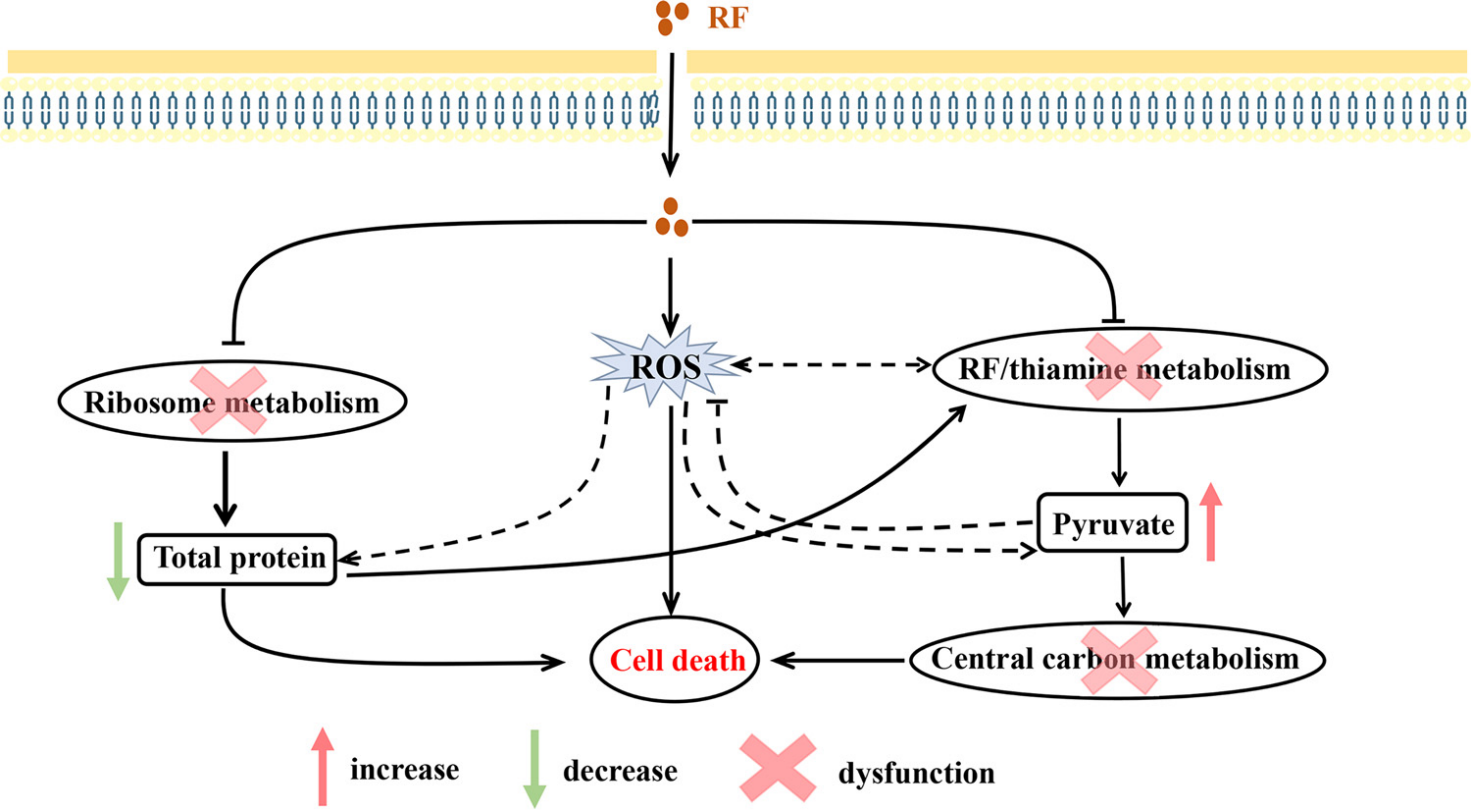
Schematic diagram of the mechanism of action of RF against C. albicans *in vitro*. Exogenous RF destroys the membrane and cell wall integrity, increasing the accumulation of ROS in C. albicans. Meanwhile, exogenous RF targets the coenzyme (including endogenous RF and thiamine metabolism) and ribosome metabolism, resulting in total metabolic disorders. These effects together lead to the death of C. albicans. Specifically, repression of endogenous RF and thiamine metabolism caused by exogenous RF induces the accumulation of pyruvate (red arrow), which therefore affects central carbon metabolism. Exogenous RF induces ribosome metabolism disorders, inducing a block in protein synthesis (green arrow). This further aggravates the disorder in thiamine metabolism, since histidine is the substrate for thiamine synthesis. Moreover, ROS are capable of inducing protein damage and interacting with endogenous RF and thiamine metabolism (35, 48–51). The accumulating pyruvate may defend against ROS (36). However, in the presence of exogenous RF, the relationship between ROS and endogenous RF or thiamine metabolism, proteins, and pyruvate (dashed line) requires further study.

Low doses of ROS are signaling molecules involved in the normal physiological functions and development of fungi, while excessive ROS doses can lead to pathological processes or cell death through their toxic effects (47). In this study, RF disrupted the membrane and cell wall integrity, resulting in the ROS accumulation. Although mitochondria are one of the main sources of ROS production, our investigation found that RF did not affect the mitochondrial respiratory chain (Fig. S4). The production of ROS may connect total metabolism via thiamine metabolism, RF metabolism, and ribosome metabolism caused by exogenous RF (Fig. 8) (48–51). More specifically, exogenous RF targets the thiamine and RF metabolic pathways, thus disrupting central carbon metabolism. The production of ROS was accompanied by an accumulation of pyruvate, which is consistent with previous work (36, 52). Ribosome metabolism was also affected by exogenous RF via the production of ROS. These data were confirmed by the downregulation of ribosome-related genes and a reduction in the total protein concentration (Fig. 6). Moreover, our findings were consistent with those of a previous investigation which showed the damaging effect of ROS on the structure and activity of proteins (50, 53). Thus, the accumulation of ROS induced by RF may contribute to the dysfunction of total metabolism. Taken together, our biochemical and transcriptome results revealed the multiple mechanisms of action of RF against C. albicans, leading to damage to the membrane and cell wall integrity, the accumulation of ROS, disordered central carbon metabolism, and protein damage (Fig. 8).

Central carbon metabolism plays an important role in fungal cells in obtaining energy and sustaining their survival. Our results suggested that exogenous RF may target endogenous thiamine and RF metabolism, resulting in a central carbohydrate metabolic disorder. Moreover, the growth of RF and thiamine metabolism-repressed C. albicans was not restored by the addition of extra RF or TPP, and extra RF or TPP had only a minor inhibitory effect on C. albicans (Fig. 5E). This was contrary to previous results showing that strains mutated in the RF or thiamine metabolic pathways would recover growth after supplementation with RF or thiamine (54, 55). Based on our findings, we speculated that exogenous RF may influence a key factor in the vitamin regulatory system (including endogenous synthesis and exogenous uptake) of C. albicans. However, further investigations are needed to confirm this key factor. Importantly, these results indicated that the endogenous RF and thiamine metabolic pathways could provide favorable targets for the development of antimicrobial drugs. Reasons for this include that thiamine and RF metabolism specifically exists in pathogens and does not exist in humans (14), and these metabolic pathways are essential for pathogen virulence (56). In fact, other B-group vitamins are important cofactors for fungal cells, including niacin (VB_3_), pantothenic acid (VB_5_), pyridoxine (VB_6_), biotin (VB_7_), folate (B_9_), and cobalamin (VB_12_), which are also potential candidates for the development of antifungal drugs (56).

*In vivo*, the inhibitory effect of RF on C. albicans virulence factors, including hyphae and biofilms, was also observed in the phenotype of tongues and histopathological analysis in an OPC mouse model. Additionally, the results of histopathological analysis and fungal burden qualitatively confirmed that the infection of mice was reduced by RF (Fig. 7). Interestingly, the effective concentration of RF (0.125 or 1 mg/kg) *in vivo* is much lower than that of FCZ (13.6 mg/kg). RF treatment showed a 2-order-of-magnitude reduction in kidney fungal burden, which was superior to the 1-order-of-magnitude reduction in the tongue. The metabolism of RF is inextricably linked to the kidney (12), suggesting that understanding the absorption and transport of RF is vital to studying the mechanism of anti-infection activity *in vivo*. In a mouse ulcer model, RF alleviated oral ulcers by exerting antioxidant and antibacterial activity and reducing the local secretion of inflammatory factors (57). Previously, 20 to 100 mg/kg of RF combined with azithromycin was shown to reduce the expression of tumor necrosis factor alpha (TNF-α), gamma interferon (IFN-γ), interleukin-6 (IL-6), and monocyte chemoattractant protein 1 (MCP-1) in S. aureus infection (58). In addition, mucosa-associated invariant T cells are reportedly involved in the pathogenesis of oral diseases and can be activated by RF in an MR1-dependent manner (59). These data imply that RF may play a strong role in immune regulation *in vivo*. However, the mechanism of action of the direct anti-*Candida* effect and the immune reaction to RF in the OPC mouse model require further investigation.

In summary, the present study reveals the anti-*Candida* effect of RF *in vitro* and *in vivo*. Explorations of the anti-*Candida* mechanisms *in vitro* confirmed a multitarget effect. Among the repressed metabolic pathways, the thiamine and RF metabolic pathways were uniquely affected by the action of RF, which provides insight into the metabolic targets of RF.

## MATERIALS AND METHODS

### Strains, media, and chemicals.

Nine *Candida* species were used in this study. Standard strains C. albicans ATCC MYA-2876, C. albicans ATCC 90028, C. parapsilosis ATCC 22019, and C. krusei ATCC 6258 were purchased from the American Type Culture Collection (ATCC). Clinical isolates C. parapsilosis Cp1 and Cp2, Candida tropicalis Ct2 and Ct3, and Candida glabrata Cg1 were from the affiliated hospital of Southwest Medical University. RF (Sigma, Shanghai, China), RF injection (commercially available), and RPMI 1640 medium (HyClone, Chengdu, China) were purchased as reagents. Fungal cells were cultured and maintained in YPD medium (1% yeast extract, 2% peptone, and 2% glucose).

### Antifungal susceptibility assay.

According to CLSI standard M27-A3, a microdilution broth susceptibility assay was conducted to determine the MIC (60). Briefly, the activated yeast cells were adjusted to 0.5 × 10^4^ to 2.5 × 10^4^ cells/mL and 100 μL of yeast suspension was added into 96-well plates. To each well, 100 μL of working solution ranging from 0.0 to 1.6 mg/mL of RF (diluted in RPMI 1640 medium) was added. The plate was incubated for 24 h at 37°C, and then the optical density at 600 nm (OD_600_) was measured. The MIC that suppressed fungal growth by 90% was defined as the lowest concentration.

Next, a spot assay determined the inhibitory activity on the solid medium. The activated cells were adjusted to 1 × 10^2^ to 3 × 10^2^, 10^3^, and 10^4^ cells/mL, and then 3 μL was spotted onto YPD agar, which contained 0.1, 0.2, 0.3, or 0.4 mg/mL RF. Phosphate-buffered saline (PBS) was added to the YPD plate as a control. After incubation for 48 h at 37°C, images of the colonies were recorded using a digital camera.

### Growth curve assay.

The strains grown overnight were diluted to 5 × 10^5^ cells/mL in YPD medium, containing 0.4 mg/mL of RF or 64 μg/mL of FCZ. The control was free of RF and FCZ. The cells were incubated at 37°C with constant shaking (200 rpm). At 0, 2, 4, 8, 12, 16, and 24 h, cells were harvested and washed with PBS before being spread onto YPD agar medium. Samples supplemented with water were considered controls. The fungal CFU were determined after being incubated for 24 h at 37°C.

### Hyphal formation.

The activated yeast cells were adjusted to a final concentration of 1 × 10^6^ to 3 × 10^6^ cells/mL in RPMI 1640 plus 10% (vol/vol) fetal bovine serum (FBS) liquid medium or YPD plus 10% (vol/vol) FBS solid medium containing 1× MIC or 2× MIC of RF. Sterile water was added as a control group. Liquid and solid media were incubated for 6 h and 5 days at 37°C, respectively.

### Biofilm assay.

Biofilm susceptibility was measured by CV and XTT assays (61). In brief, the activated yeast cells were suspended in RPMI 1640 (1 × 10^6^ to 3 × 10^6^ cells/mL) and 200 μL was added to 96-well plates. For the initial phase, cultures were preincubated for 1.5 h and RF was added for 6 h at 37°C. Then, the supernatant was removed and incubation was continued for 45.5 h. For the developmental phase, cultures were preincubated for 12 h and RF was added for 6 h at 37°C. Then, the supernatant was removed and incubation was continued for 36 h. For the maturation phase, cultures were preincubated for 48 h and RF was added for 6 h at 37°C. The biofilm biomass and activity were measured by the CV and XTT methods, as follows. (i) For the CV method, 100 μL of 15% CV was added to each well and incubated at room temperature for 10 min. After washing with PBS, 200 μL of 15% acetic acid was added, and after 30 min at room temperature, the OD_600_ was measured. (ii) For the XTT method, 1 mg/mL of XTT (in PBS) and 0.4 mM menadione (in ethanol) were prepared in a 5:1 ratio. To each well, 200 μL PBS and 12 μL XTT-menadione solution were added, and after 2 h of incubation at 37°C in the dark, the OD_490_ was measured.

### Confocal laser scanning microscopy.

The biomass and three-dimensional structure of the biofilm were analyzed by CLSM (62). The activated fungal cells were resuspended in fresh RPMI 1640 at a final concentration of 1 × 10^6^ to 3 × 10^6^ cells/mL. Then, 3 mL of fungal cells was added to 6-well plates with circular microscope cover glasses in the bottom of the wells. After incubation for 90 min at 37°C, RF was added to a final concentration of 1× MIC and continuously incubated for 6 h. For the control groups, sterile water was added. Subsequently, plates were washed with PBS and samples were incubated with fresh RPMI 1640 medium for 24 h at 37°C. The biofilm was washed with PBS and stained with 1 mL of CFW for 3 min in the dark at room temperature. The circular microscope cover glasses were then transferred into antifade mounting medium, before being placed upside down on a glass slide, and the biofilm was observed through a TCS SP8 CLSM.

### Cell wall assay to determine cell wall integrity and glucan and chitin content.

The fungal cells were treated with 1× MIC or 2× MIC of RF for 12 h at 37°C with shaking at 200 rpm. Sterile water was added as a control. Cells were harvested and washed with PBS, and then the fungal suspension was treated in three ways. (i) To the fungal suspension, 3 μL of 1% KOH and 7 μL of CFW were added, and after 2 to 3 min in the dark at room temperature, the cell morphology was observed through a DP80 fluorescence microscope (Olympus, Tokyo, Japan). (ii) The fungal suspension was adjusted to 1 × 10^2^ to 3 × 10^2^, 1 × 10^3^, and 1 × 10^4^ cells/mL and then spotted onto YPD agar medium containing 50 μg/mL of CFW. After being incubated for 5 days at 37°C, the colonies were recorded using a digital camera. (iii) The fungal suspension was adjusted to 5 × 10^7^ cells/mL, and then the OD_600_ was measured. To determine the chitin content, CFW (at a final concentration of 3.5 μg/mL) was added and staining was carried out at 37°C for 10 min. The fluorescence intensity was measured at 365-nm excitation and 435-nm emission wavelengths on a BioTek Varioskan Synergy H1 plate reader (Thermo Fisher Scientific, Shanghai, China). To determine the total glucan content, aniline blue (1%) was added and incubated at 80°C for 15 min in the dark. Fluorescence intensity was measured at 398-nm excitation and 508-nm emission wavelengths.

### PI staining.

The cell membrane integrity was determined by PI (Solarbio, Beijing, China) staining (60), an ergosterol content assay, and gene expression analysis. Sample collection was the same as for the cell wall integrity assay. The cells were resuspended to 0.5 × 10^7^ to 1.0 × 10^7^ cells/mL. Then, PI (10 μg/mL) was added and incubated for 30 min at 37°C in the dark, followed by PBS washing. The cell membrane permeability was determined using a FACSAria flow cytometer (BD Biosciences, NJ, USA) and an inverted fluorescence microscope. The blank group was not treated with PI. The positive-control group was pretreated with hydrogen peroxide for 20 min at 37°C.

### HPLC assay.

The ergosterol content of *Candida* was evaluated by high-performance liquid chromatography (HPLC) (1260 Infinity II; Agilent). In brief, after centrifuging and washing, the wet samples were adjusted to 0.5 g. Then, 25 mL of methanol was added to samples and the total weight was recorded, followed by an ultrasound for 1 h. Following cooling to room temperature, methanol was added to the original weight and the solution was filtered through a 0.45-μm Millipore filter and stored at −20°C. The ergosterol was determined at 283 nm by HPLC.

### ROS level measurement.

The fresh fungal cells (1 × 10^6^ cells/mL) were treated with 1× MIC or 2× MIC RF for 6 h at 37°C with constant shaking (200 rpm). Samples were resuspended to 0.5 × 10^7^ to 1.0 × 10^7^ cells/mL in PBS and stained with 10 μM dichlorodihydrofluorescein diacetate (DCFH-DA) (Sigma, Shanghai, China) for 30 min at 37°C. After centrifuging and removing excess DCFH-DA, cells were resuspended. A FACSAria flow cytometer and inverted fluorescence microscope were used to analyze the ROS levels.

### Spot assay.

The fresh fungal cells (1 × 10^6^ cells/mL) were treated with 1× MIC or 2× MIC RF for 12 h at 37°C with constant shaking (200 rpm). Cells were washed with PBS and diluted over a gradient. To investigate the effect of RF on C. albicans RF and thiamine metabolism, 3 μL of fungal suspension was spotted onto the YPD agar (containing RF or thiamine pyrophosphate) and cultured for 2 to 4 days at 37°C. To assess the effect of RF on the ability for carbon source utilization, a gradient dilution of the fungal suspension was spotted onto YPD agar (glucose source) or glycerol agar (1% yeast extract, 2% peptone, 2% glycerol, and 2% agar), and cultured for 2 to 4 days at 37°C.

### RNA sequencing.

C. albicans ATCC MYA-2876 (1 × 10^7^ cells/mL) was treated with 1× MIC RF or without RF for 12 h at 37°C with constant shaking (200 rpm). Samples were collected by centrifugation for 2 min at 12,000 × *g* and 4°C. Then, total RNA was isolated using yeast processing reagent (TaKaRa, Dalian, China). The transcriptome data were processed by Biomarker (Beijing and Qingdao, China) using Oxford Nanopore Technologies Long Read Processing (63). The standard for the screening of differentially expressed genes was a |fold change| of ≥2 and a false-discovery rate of ≤0.01.

### RT-qPCR analysis.

The activated C. albicans ATCC MYA-2876 was adjusted to 1 × 10^7^ cells/mL and grown in YPD liquid medium at 37°C with shaking at 200 rpm. Samples were collected at 0, 6, and 12 h. Total RNA was isolated as previously described. The PrimeScript RT reagent kit with genomic DNA (gDNA) eraser and TB Green Premix *Ex Taq* II (TaKaRa, Beijing, China) were used to generate reverse cDNA and for RT-qPCR. The transcript of the β-actin (*ACT1*) gene was used as an internal standard. All primers are shown in Table S1 in the supplemental material. The threshold cycle (2^−ΔΔ^*^CT^*) method was used to determine the relative change in gene expression (64).

### Total protein assay.

C. albicans ATCC MYA-2876 (1 × 10^7^ cells/mL) was treated with 1× MIC or 2× MIC RF or without RF for 12 h at 37°C with constant shaking (200 rpm). Samples were collected by centrifugation, washed three times, and adjusted to 1 × 10^8^ cells/mL. Total protein was collected by a yeast total protein extraction kit (Sangon Biotech, Shanghai, China). Subsequently, the protein level was tested by an enhanced bicinchoninic acid (BCA) protein assay kit (Beyotime, Shanghai, China).

### Quantification of the pyruvate concentration.

C. albicans ATCC MYA-2876 was cultured as described for the total protein assay. Samples were adjusted to 2 × 10^7^ cells/mL and 5 × 10^7^ cells/mL for the determination of pyruvate and acetyl coenzyme A (acetyl-CoA), respectively. Samples were collected by centrifugation and ground with liquid nitrogen, and then the concentration of pyruvate was quantified by a pyruvic acid content assay kit (Boxbio, Beijing, China).

### Antifungal effect *in vivo*.

All experimental protocols were approved by the Southwest Medical University Institutional Animal Care and Use Committee (2020540). C57BL/6J male mice (6 to 8 weeks old; SiPeiFu, Beijing, China) were fed at 25 ± 1°C for 1 week before the experiment. The mice were assigned to five groups of eight mice each. One day before infection, and on the 1st and 3rd days after infection, mice were subcutaneously injected with 200 μL of the immunosuppressant cortisone acetate (225 mg/kg), which was dissolved in normal saline containing 0.5% Tween 80 (65, 66). On the day of infection, all groups were narcotized by intraperitoneal injection with pentobarbital (50 mg/kg) and the dorsum of the tongue, free of redness and hemorrhage, was scratched with a scalpel. Subsequently, a cotton ball saturated with 100 μL of C. albicans ATCC (2 × 10^8^ cells/mL) was placed in the oral cavity for 90 min. Two hours after infection, RF (0.125 or 1 mg/kg) or FCZ (13.6 mg/kg) was intraperitoneally injected into the treatment group, and normal saline was intraperitoneally injected into the control group and the Ca (infection) group. When the intraperitoneal injection volume is 100 μL, 0.125 mg/kg and 1 mg/kg of RF correspond to 1/16× MIC and 1/2× MIC of RF. As previously described (65), 13.6 mg/kg of FCZ was used to treat oropharyngeal candidiasis in mice. The mice were injected once every 24 h for 5 consecutive days. After the last treatment, the mice were euthanized and the tongue, kidney, and liver tissues were collected for determination of the fungal load and pathological analysis.

### Statistical analysis.

The *in vitro* experiments and RNA-Seq were performed three independent experiments, except the animal experiments. The differences between the groups were compared by a *t* test or one-way analysis of variance (ANOVA) followed by a least significant difference (LSD) test or Tamhane T2 test using IBM SPSS Statistics 26 (IBM SPSS Inc., Chicago, IL, USA). *P* values of <0.05 were considered statistically significant. GraphPad Prism 9.0 software was used to generate all of the figures.

### Data availability.

Sequence data were deposited in the Beijing Institute of Genomics Genome Sequence Archive (accession no. PRJCA007860).
