# Correction for Lei et al., “Riboﬂavin Targets the Cellular Metabolic and Ribosomal Pathways of *Candida albicans In Vitro* and Exhibits Efﬁcacy against Oropharyngeal Candidiasis”

**DOI:** 10.1128/spectrum.03632-23

**Published:** 2024-03-05

**Authors:** Junwen Lei, Jian Huang, Caiyan Xin, Fangyan Liu, Jinping Zhang, Yuxin Xie, Yingyu Mao, Wenbi Chen, Zhangyong Song

## AUTHOR CORRECTION

Volume 11, no. 1, e03801-22, 2023, https://doi.org/10.1128/spectrum.03801-22. Page 3, Fig. 1A, third row: The image of *C. albicans* ATCC MYA-2876 (10^3^) was incorrectly repeated for *C. parapsilosis* ATCC 22019 (10^2^). Panel A should appear as shown below; spaces and a dashed line denote images joined from different areas of the plate.

­


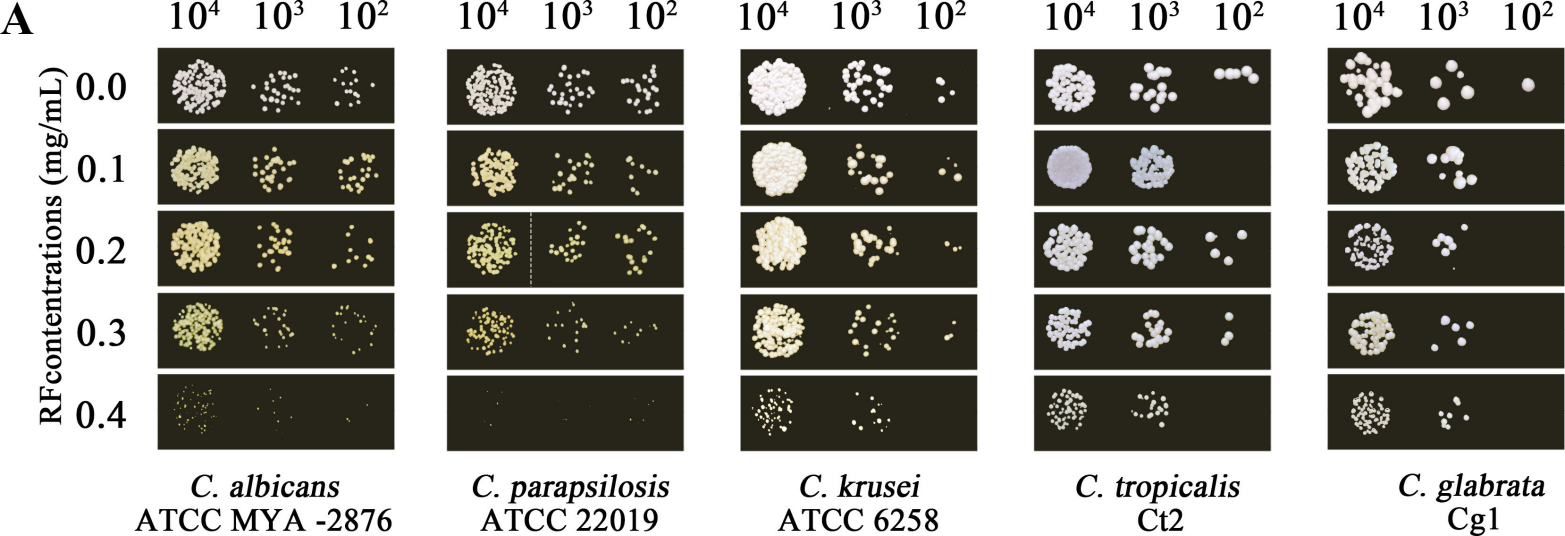


­

Page 5: The experiments corresponding to Fig. 2E and 5E were conducted simultaneously, necessitating the use of the same control group in the two figures. Figure 2E should appear as shown below; black lines divide images joined from different areas of the plate.

­


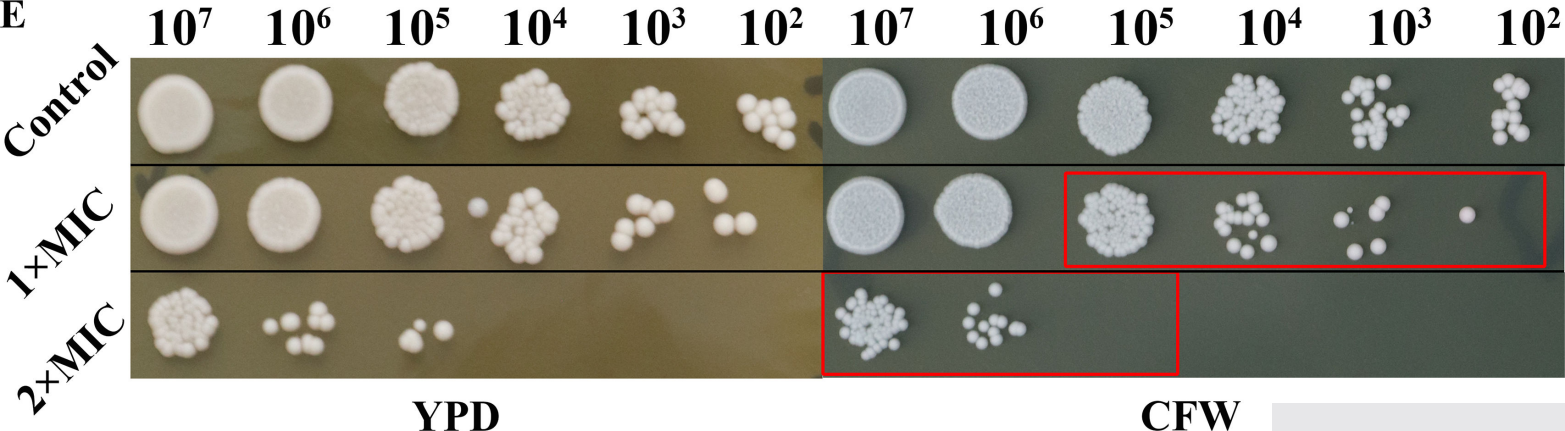


­

Page 8, Fig. 5E, first row: The panels for the Control group and the 1×MIC group were reversed. Figure 5E should appear as shown below.

­


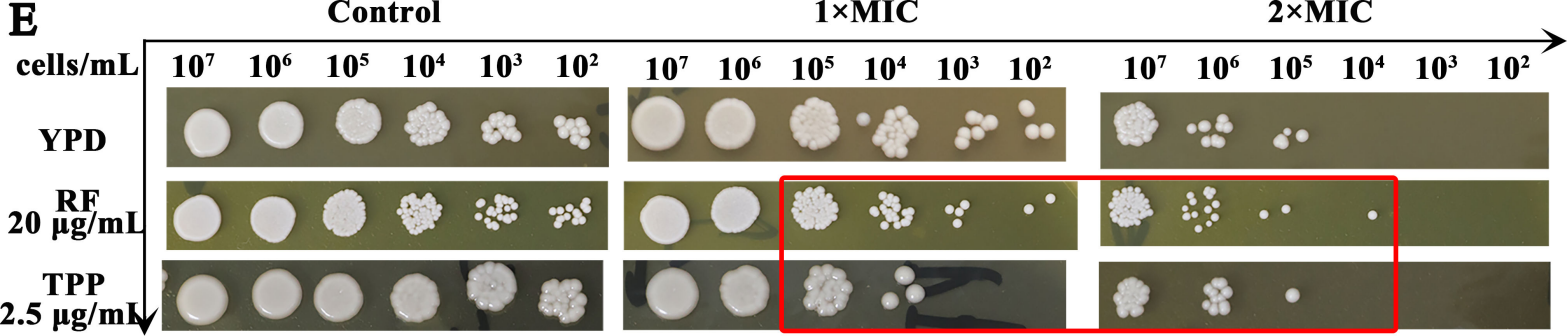


­

These corrections do not affect the overall discussion and conclusions.

