# Supplementary material for: Riboflavin Targets the Cellular Metabolic and Ribosomal Pathways of Candida albicans In Vitro and Exhibits Efficacy against Oropharyngeal Candidiasis
Source: Microbiol Spectr. 2023 Jan 10;11(1):e03801-22. doi: 10.1128/spectrum.03801-22 (PMC9927497; doi:10.1128/spectrum.03801-22)
Supplement: Supplemental file 1 — Figures S1-S4. Download spectrum.03801-22-s0001.pdf, PDF file, 0.5 MB [file spectrum.03801-22-s0001.pdf]

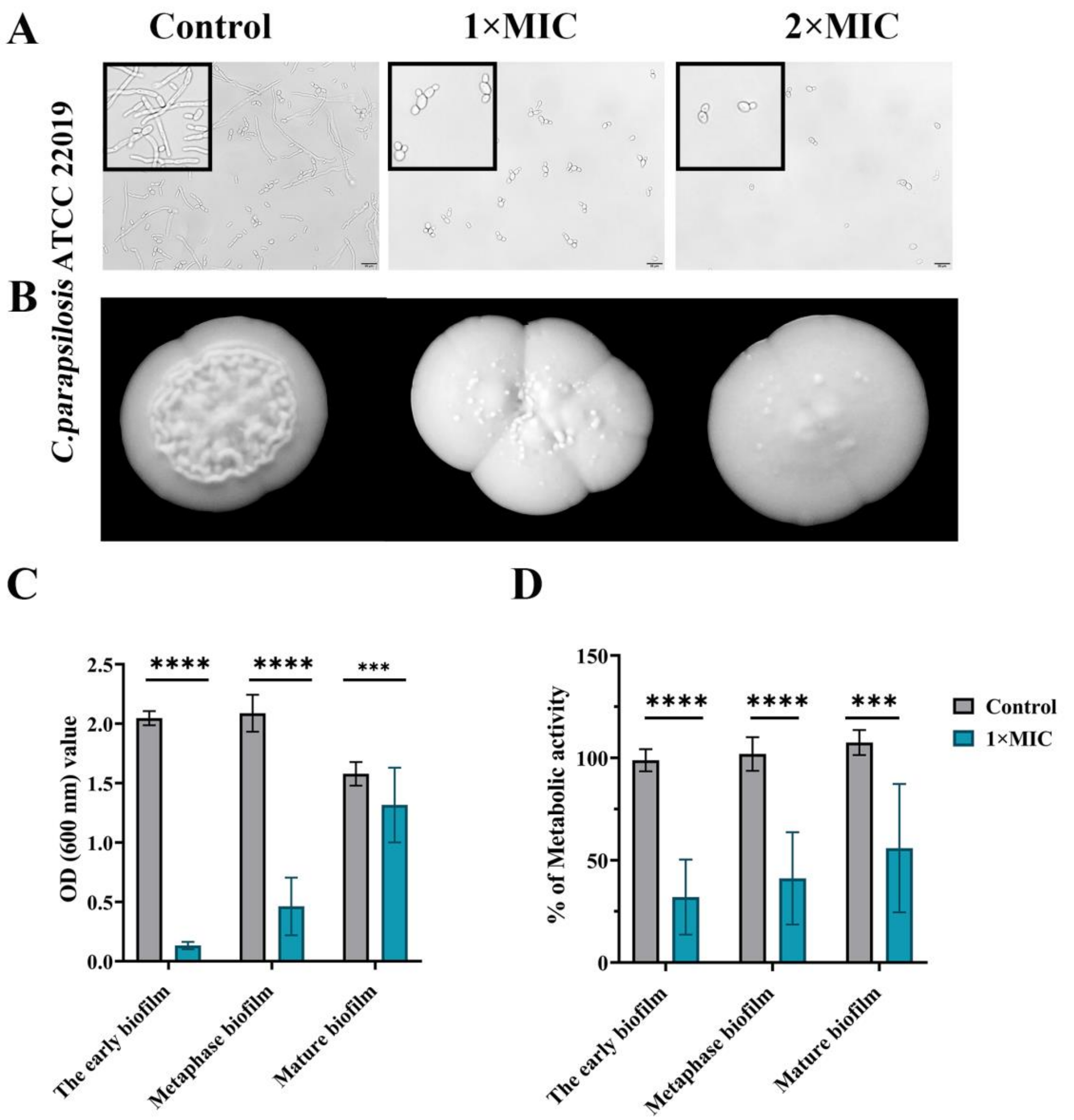

FIG S1 The effect of RF on *C. parapsilosis* hyphal and biofilm formation. (A) Hyphae formation was evaluated in a 1640 RPMI + 10 % (v/v) FBS liquid medium and the uniformly enlarged image is presented in the black boxes on the left hand. Scale bar: 20 μm. (B) Hyphae formation was evaluated on YPD+10 % (v/v) FBS agar plates. (C) The biomass of *C. albicans* biofilm was observed by a crystal violet (CV) assay. (D) Metabolic activity of *C. albicans* biofilm was determined by 2,3-bis-(2-methoxy-4-nitro-5-sulfophenyl)-2H-tetrazolium-5-carboxanilide (XTT) assay and the results are presented as relative percentages. Data were analyzed by t test (ns $P > 0.05$,* $P < 0.05$, ** $P < 0.01$, *** $P < 0.001$, **** $P < 0.0001$).

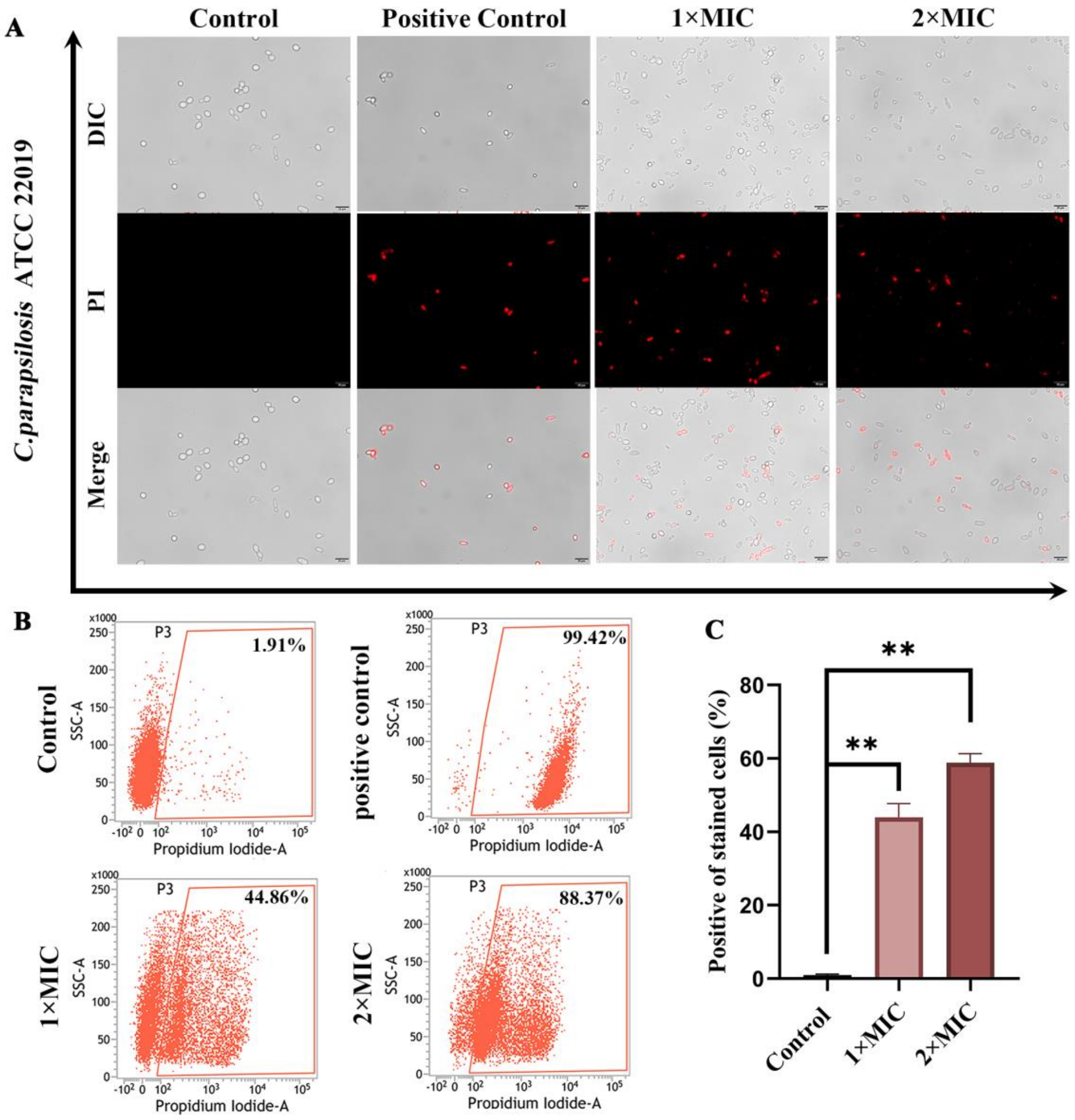

FIG S2 The effect of RF on *C. parapsilosis* cell membrane. *C. parapsilosis* cells with or without RF treatment were stained with propidium iodide (PI) and analyzed by (A) fluorescence microscopy and (B) flow cytometry. Scale bar: 20 µm. (C) Histogram analysis shows the percentage of PI-positive cells. Data were analyzed by ANOVA (ns $P > 0.05$,* $P< 0.05$, ** $P< 0.01$, *** $P< 0.001$, **** $P< 0.0001$).

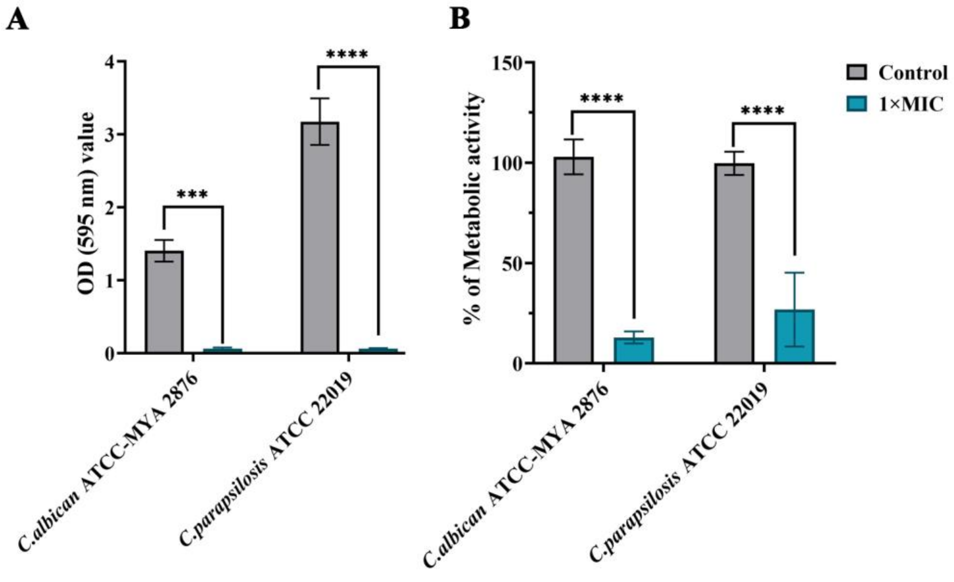

FIG S3 The effect of RF on adhesion. The adhesion of *C. albicans* and *C. parapsilosis* were examined by (A) CV assay and (B) XTT assay. Data were analyzed by t test (ns $P > 0.05$, * $P < 0.05$, ** $P < 0.01$, *** $P < 0.001$, **** $P < 0.0001$).

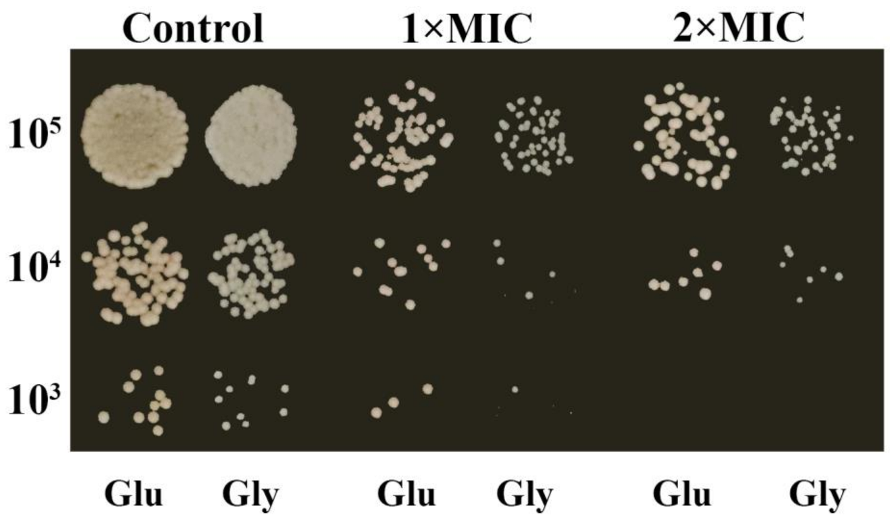

FIG S4 Effects of RF on glucose (Glu) and glycerol (Gly) utilization in *C. albicans*. Strains with defective respiratory function are unable to utilize non-fermentative carbon sources (glycerol).

TABLE S1 Primers sequence of target genes

Gene	Sequence (5'-3')
ACT1-F	CCAGGTATTGCTGAACGTATGC
ACT1-R	ACTCTGTCTGGATTGGTGGTTC
HWP1-F	TCTCTACGACTGAAGGTGCTATTC
HWP1-R	GACTTTCGGTGCTGCTATTATTGG
ALS3-F	CCTATACCACTGCTACTACCGTT
ALS3-R	CGTCCTCATTACACCAACCATAC
BCR1-F	TACACCTAAAAGAAGACCCGAACC
BCR1-R	ACCAACACCAATATGAACACGG
NDT80-F	AATCTACCCTGCAGTTCCTTCAG
NDT80-R	AGGGGAAACTACTACCAAGAGTAC
ERG11-F	TGGAGACGTGATGCTGCTCA
ERG11-R	ACGTGGTGATATTGATCCAAATCGT
ECE1-F	CCAGAAATTGTTGCTCGTGTTG
ECE1-R	CTTGTTGAACAGTTTCCAGGACG
CDA2-F	GTGGCGGCAGTGGATTAC
CDA2-R	AGGTGGATCTAAATCTGGCCATT
ERG2-F	GCCACGGCCATTATGATTGA
ERG2-R	AGTCCCAATTGCTGTACCGA
ERG11-F	TGGAGACGTGATGCTGCTCA
ERG11-R	ACGATTTGGATCAATATCACCACGT
RIB4-F	TGGTATTCTTCATGCCAGATGGA
RIB4-R	AGGTAAACCATTAGACGCCA
RIB5-F	ACTCTCCGCAGAACCAATCT
RIB5-R	TGGCCACCCAATCTAACTTCA

THI4-F	GCCACTGCCGTTGAAGATTT
THI4-R	CTTCTGGTCACGATGGACCT
THI13-F	GAGCCCTCCAATCCATCTGA
THI13-R	TGGGAGTCCTCTAGCTTTGG
PGM2-F	ACCAGGAGATTCCGTTGCTA
PGM2-R	TCCAACCAGTAGGAACTTCA
TPI1-F	ACCAAGTTTGCCTTGGACAC
TPI1-R	GGGTAGCGGCTAAACCAGTA
RHR2-F	GGATACCATGACGCCCACTA
RHR2-R	GCACCTTTACCTGCGGTTAT
MLS1-F	AGAAACAGAGCTGCTTTGGC
MLS1-R	TGGTTCCTTTGGTGGCAATG
PCK1-F	CTTGGTGTTGCACCCAATGA
PCK1-R	AGAACCAACCCAACCAGTGT
FDH1-F	AGAGGTATCGCTGCCATTGA
FDH1-R	AACAGCAGCAACATCCCAAG
ADH2-F	TTAGAAGCCGGTCAATGGGT
ADH2-R	CATGGCCTTGGCGTATTGAA
RPL3-F	ACGTCACGGTTCCTTAGGTT
RPL3-R	AAGGCAGTCAAAGCAACTGG
RPL4B-F	AAGGCAGTCAAAGCAACTGG
RPL4B-R	GCTTGTTCAACCTTGGCAGA
RPL10-F	AGGTATGAGAGGTGCTTGGG
RPL10-R	TTGTTGACCTGGGAACTTGT
RPL17B-F	CCTGCTAACCCAGCCAAATC
RPL17B-R	TGGCTTGAGCAGTTTCTCTG
RPP1A-F	TCAACTTCTCTGCTGCTCCA
RPP1A-R	TTCTTCAGCAGCTTCTTCGG
YST1-F	CCCAAGAACCGATGCTCAAG
YST1-R	TCTCTAGCAAGCAACCACCA
RPS14B-F	TTGCCAGAGTTACTGGTGGT
RPS14B-R	AGCTCTTAAAGCGGATTGACC
ASC1-F	CGCTGACTTCATTGGTCACA
ASC1-R	AAGCATGAACTTCGGCCTTG
